# Ethyl 3-[1-(4-methoxy­phen­yl)-4-oxo-3-phenylazetidin-2-yl]-2-nitro-1-phenyl-2,3,10,10a-tetra­hydro-1*H*,5*H*-pyrrolo[1,2-*b*]isoquinoline-10a-carboxyl­ate

**DOI:** 10.1107/S1600536810005696

**Published:** 2010-02-17

**Authors:** S. S. Sundaresan, P. Ramesh, N. Arumugam, R. Raghunathan, M. N. Ponnuswamy

**Affiliations:** a Centre of Advanced Study in Crystallography and Biophysics, University of Madras, Guindy Campus, Chennai 600 025, India; b Department of Organic Chemistry, University of Madras, Guindy Campus, Chennai 600 025, India

## Abstract

In the title mol­ecule, C_37_H_35_N_3_O_6_, the pyrrolidine ring adopts a twist conformation and the piperidine ring is in a distorted boat conformation. One of the phenyl rings is disordered over two positions with occupancies of 0.54 (2) and 0.46 (2) and the ethyl carboxyl­ate group is also disordered over two orientations with occupancies of 0.75 (1) and 0.25 (1).

## Related literature

For the pharmacological properties of β-lactam derivatives, see: Jones *et al.* (1989 ▶); Page (1992 ▶); Hashimoto *et al.* (1976 ▶); Bose *et al.* (1974 ▶); Fujisawa *et al.* (1995 ▶); Han *et al.* (1995 ▶); Adlington *et al.* (1997 ▶); Borthwick *et al.* (1998 ▶); Palomo *et al.* (1999 ▶); Kamel & Naser (1979 ▶). For puckering and asymmetry parameters, see: Cremer & Pople (1975 ▶); Nardelli *et al.* (1983 ▶). For hybridization, see: Beddoes *et al.* (1986 ▶).
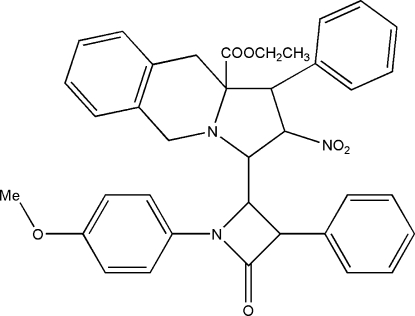


## Experimental

### 

#### Crystal data

C_37_H_35_N_3_O_6_
*M* *_r_* = 617.68
Triclinic, 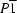

*a* = 9.3039 (3) Å
*b* = 13.0725 (3) Å
*c* = 13.8814 (3) Å
α = 87.504 (1)°
β = 74.123 (1)°
γ = 74.926 (1)°
*V* = 1567.35 (7) Å^3^
*Z* = 2
Mo *K*α radiation
μ = 0.09 mm^−1^
*T* = 293 K
0.20 × 0.20 × 0.17 mm

#### Data collection

Bruker Kappa APEXII area-detector diffractometer
Absorption correction: multi-scan (*SADABS*; Sheldrick, 2001 ▶) *T*_min_ = 0.982, *T*_max_ = 0.985
30422 measured reflections
5887 independent reflections
4326 reflections with *I* > 2σ(*I*)
*R*_int_ = 0.029

#### Refinement

*R*[*F* ^2^ > 2σ(*F* ^2^)] = 0.044
*wR*(*F* ^2^) = 0.121
*S* = 1.05
5887 reflections
490 parameters
97 restraints
H-atom parameters constrained
Δρ_max_ = 0.19 e Å^−3^
Δρ_min_ = −0.20 e Å^−3^

### 

Data collection: *APEX2* (Bruker, 2004 ▶); cell refinement: *SAINT* (Bruker, 2004 ▶); data reduction: *SAINT*; program(s) used to solve structure: *SHELXS97* (Sheldrick, 2008 ▶); program(s) used to refine structure: *SHELXL97* (Sheldrick, 2008 ▶); molecular graphics: *ORTEP-3* (Farrugia, 1997 ▶); software used to prepare material for publication: *SHELXL97* and *PLATON* (Spek, 2009 ▶).

## Supplementary Material

Crystal structure: contains datablocks global, na325, I. DOI: 10.1107/S1600536810005696/ci2997sup1.cif

Structure factors: contains datablocks I. DOI: 10.1107/S1600536810005696/ci2997Isup2.hkl

Additional supplementary materials:  crystallographic information; 3D view; checkCIF report

## Figures and Tables

**Fig. 1.**
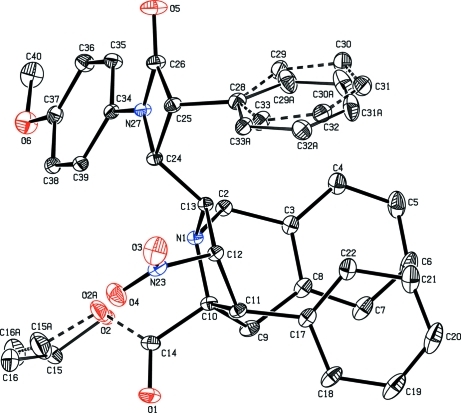
The molecular structure of the title compound, showing displacement ellipsoids drawn at the 30% probability level. All disorder components are shown. H atoms have been omitted for clarity.

## supplementary crystallographic information

### Comment

β-lactam antibiotics have been successfully used in the treatment of infectious diseases for many years (Jones *et al.*, 1989). Despite the large number of compounds containing a β-lactam moiety that have already been synthesized and tested, there is still a need for new compounds of this kind due to the increasing resistance of bacterial strains to certain types of antibiotics (Page, 1992). A class of β-lactam known as the monocyclic β-lactam, which includes compounds such as the nocardicins, aztreonam and carumonam, has been described for their chemotherapeutic importance as antibiotics (Hashimoto *et al.*, 1976; Bose *et al.*, 1974; Fujisawa *et al.*, 1995). The recent discovery of new biologically active monocyclic β-lactam compounds displaying activities other than the usual antibiotic one, such as thrombin (Han *et al.*, 1995), prostate-specific antigen (Adlington *et al.*, 1997), human cytomegalovirus protease (Borthwick *et al.*, 1998) and the cholesterol absorption inhibitors (Palomo *et al.*, 1999), are also interesting. The presence of a carbohydrate side chain in a drug may also overcome the frequently observed water insolubility problem (Kamel & Naser, 1979).

The pyrrolidine ring in the title molecule (Fig. 1) adopts a twist conformation, with puckering (Cremer & Pople, 1975) and asymmetry parameters (Nardelli, 1983) of q_2_ = 0.275 (2) Å, φ = 92.8 (3)° and Δ_2_(N1) = 2.8 (2)°. The sum of angles around N1 (339.53°) is in accordance with *sp*^3^ hybridization (Beddoes *et al.*, 1986). The piperidine ring adopts a distorted boat conformation with the puckering and asymmetry parameters q_2_ = 0.641 (2) Å, q_3_ = -0.005 (2) Å, φ_2_ = 64.3 (2)° and Δ_s_(C2,C9) = 4.7 (2)°. The β-lactam ring is planar and the keto atom O5 deviates from this plane by -0.054 (2) Å. The methoxy group is slightly twisted out of the attached C34–C39 phenyl ring [C40—O6—C37—C36 = 5.4 (3)°].

A weak intermolecular C—H···π interaction involving the C9–H9B group and the C3–C8 benzene ring (centroid Cg1) of the molecule at (1-x, 1-y, 1-z) is observed [H9B···Cg1 = 2.95 Å, C9···Cg1 = 3.889 (2) Å and C9···H9B···Cg1 = 163°].

### Experimental

To a stirred solution of 5-(1'-*N*-(*p*-methoxyphenyl-azetidine-2'-one)-4-nitro-3-phenyl-2-ethoxycarbonyl-2-benzyl-pyrrlolidine (1 mmol) in dry chloroform (20 ml) was added *p*-formaldehyde (1 mmol) followed by trifluroacetic acid (0.1 mmol) at room temperature. After completion of the reaction, the mixture was washed with water and dried over Na_2_SO_4_. The solvent was removed under the reduced pressure and the crude product was subjected to column chromatography with hexane-ethyl acetate (9:1)to obtain pure cyclized product. The compound was recrystallized from ethyl acetate.

### Refinement

One of the phenyl rings is disordered over two positions with occupancies of 0.54 (2) and 0.46 (2) and the ethyl carboxylate group is also disordered over two orientations with occupancies of 0.753 (10) and 0.247 (10). The C—C distances in the disordered components were restrained to be equal and U_ij_ parameters of atoms C15A, C16A, C32, C32A and C33A were restrained to an approximate isotropic behaviour. All H atoms were positioned geometrically (C-H = 0.93–0.98 Å) and allowed to ride on their parent atoms, with *U*_iso_(H) = 1.5*U*_eq_(C) for methyl H and 1.2*U*_eq_(C) for other H atoms. The reflection '0 1 0' affected by beamstop was removed during refinement.

### Crystal data

**Table:**

C_37_H_35_N_3_O_6_	*Z* = 2
*M**_r_* = 617.68	*F*(000) = 652
Triclinic, *P*1	*D*_x_ = 1.309 Mg m^−^^3^
Hall symbol: -P 1	Mo *K*α radiation, λ = 0.71073 Å
*a* = 9.3039 (3) Å	Cell parameters from 3651 reflections
*b* = 13.0725 (3) Å	θ = 1.5–25.6°
*c* = 13.8814 (3) Å	µ = 0.09 mm^−^^1^
α = 87.504 (1)°	*T* = 293 K
β = 74.123 (1)°	Block, colourless
γ = 74.926 (1)°	0.20 × 0.20 × 0.17 mm
*V* = 1567.35 (7) Å^3^	

### Data collection

**Table:**

Bruker Kappa APEXII area-detector diffractometer	5887 independent reflections
Radiation source: fine-focus sealed tube	4326 reflections with *I* > 2σ(*I*)
graphite	*R*_int_ = 0.029
ω and φ scans	θ_max_ = 25.6°, θ_min_ = 1.5°
Absorption correction: multi-scan (*SADABS*; Sheldrick, 2001)	*h* = −11→11
*T*_min_ = 0.982, *T*_max_ = 0.985	*k* = −15→15
30422 measured reflections	*l* = −16→16

### Refinement

**Table:**

Refinement on *F*^2^	Primary atom site location: structure-invariant direct methods
Least-squares matrix: full	Secondary atom site location: difference Fourier map
*R*[*F*^2^ > 2σ(*F*^2^)] = 0.044	Hydrogen site location: inferred from neighbouring sites
*wR*(*F*^2^) = 0.121	H-atom parameters constrained
*S* = 1.05	*w* = 1/[σ^2^(*F*_o_^2^) + (0.0504*P*)^2^ + 0.3689*P*] where *P* = (*F*_o_^2^ + 2*F*_c_^2^)/3
5887 reflections	(Δ/σ)_max_ = 0.001
490 parameters	Δρ_max_ = 0.19 e Å^−^^3^
97 restraints	Δρ_min_ = −0.20 e Å^−^^3^

### Special details

**Table:**

Geometry. All e.s.d.'s (except the e.s.d. in the dihedral angle between two l.s. planes) are estimated using the full covariance matrix. The cell e.s.d.'s are taken into account individually in the estimation of e.s.d.'s in distances, angles and torsion angles; correlations between e.s.d.'s in cell parameters are only used when they are defined by crystal symmetry. An approximate (isotropic) treatment of cell e.s.d.'s is used for estimating e.s.d.'s involving l.s. planes.
Refinement. Refinement of *F*^2^ against ALL reflections. The weighted *R*-factor *wR* and goodness of fit *S* are based on *F*^2^, conventional *R*-factors *R* are based on *F*, with *F* set to zero for negative *F*^2^. The threshold expression of *F*^2^ > σ(*F*^2^) is used only for calculating *R*-factors(gt) *etc*. and is not relevant to the choice of reflections for refinement. *R*-factors based on *F*^2^ are statistically about twice as large as those based on *F*, and *R*- factors based on ALL data will be even larger.

### Fractional atomic coordinates and isotropic or equivalent isotropic displacement parameters (Å^2^)

**Table:**

	*x*	*y*	*z*	*U*_iso_*/*U*_eq_	Occ. (<1)
O1	−0.00074 (16)	0.59615 (13)	0.42249 (12)	0.0806 (4)	
O2	0.2057 (6)	0.4922 (4)	0.3151 (4)	0.0612 (9)	0.753 (10)
O2A	0.180 (2)	0.5220 (14)	0.2887 (16)	0.089 (5)	0.247 (10)
O3	0.0627 (2)	0.86406 (15)	0.16584 (14)	0.1081 (6)	
O4	0.0583 (2)	0.71036 (16)	0.22020 (13)	0.0956 (5)	
O5	0.74560 (17)	0.70097 (11)	−0.04187 (12)	0.0897 (5)	
O6	0.79171 (19)	0.19925 (11)	0.10468 (13)	0.0877 (5)	
N1	0.37346 (14)	0.63265 (10)	0.29712 (9)	0.0413 (3)	
N23	0.10037 (17)	0.79028 (15)	0.21643 (11)	0.0595 (4)	
N27	0.57227 (15)	0.63357 (10)	0.08244 (10)	0.0476 (3)	
C2	0.52195 (18)	0.58959 (13)	0.32089 (13)	0.0474 (4)	
H2A	0.6053	0.5980	0.2639	0.057*	
H2B	0.5358	0.5144	0.3324	0.057*	
C3	0.5315 (2)	0.64295 (14)	0.41105 (14)	0.0530 (4)	
C4	0.6528 (2)	0.68215 (17)	0.41500 (18)	0.0738 (6)	
H4	0.7354	0.6785	0.3584	0.089*	
C5	0.6517 (3)	0.7269 (2)	0.5032 (2)	0.1005 (9)	
H5	0.7339	0.7536	0.5062	0.121*	
C6	0.5304 (4)	0.7323 (2)	0.5864 (2)	0.1026 (10)	
H6	0.5316	0.7616	0.6459	0.123*	
C7	0.4075 (3)	0.69511 (18)	0.58325 (16)	0.0797 (7)	
H7	0.3250	0.6996	0.6402	0.096*	
C8	0.4063 (2)	0.65081 (14)	0.49505 (13)	0.0580 (5)	
C9	0.2777 (2)	0.61098 (15)	0.48137 (13)	0.0582 (5)	
H9A	0.1874	0.6356	0.5376	0.070*	
H9B	0.3069	0.5341	0.4808	0.070*	
C10	0.23601 (18)	0.64853 (13)	0.38286 (12)	0.0462 (4)	
C11	0.14835 (19)	0.76820 (13)	0.38416 (12)	0.0478 (4)	
H11	0.0381	0.7724	0.3963	0.057*	
C12	0.20748 (18)	0.80076 (13)	0.27678 (12)	0.0457 (4)	
H12	0.2140	0.8744	0.2768	0.055*	
C13	0.37003 (17)	0.72713 (12)	0.23744 (11)	0.0410 (4)	
H13	0.4435	0.7621	0.2517	0.049*	
C14	0.1301 (2)	0.57983 (17)	0.37252 (15)	0.0599 (5)	
C15	0.1191 (4)	0.4158 (4)	0.3140 (3)	0.0778 (14)	0.753 (10)
H15A	0.0584	0.4087	0.3818	0.093*	0.753 (10)
H15B	0.1908	0.3474	0.2916	0.093*	0.753 (10)
C16	0.0156 (6)	0.4446 (4)	0.2489 (3)	0.1046 (17)	0.753 (10)
H16A	−0.0397	0.3915	0.2516	0.157*	0.753 (10)
H16B	0.0753	0.4495	0.1813	0.157*	0.753 (10)
H16C	−0.0566	0.5118	0.2712	0.157*	0.753 (10)
C16A	0.1254 (17)	0.3554 (13)	0.2817 (13)	0.115 (5)	0.247 (10)
H16D	0.0578	0.3184	0.2657	0.173*	0.247 (10)
H16E	0.1264	0.3442	0.3503	0.173*	0.247 (10)
H16F	0.2282	0.3290	0.2388	0.173*	0.247 (10)
C15A	0.071 (2)	0.4669 (10)	0.2668 (19)	0.133 (7)	0.247 (10)
H15C	−0.0316	0.4946	0.3111	0.159*	0.247 (10)
H15D	0.0660	0.4787	0.1982	0.159*	0.247 (10)
C17	0.1640 (2)	0.84124 (14)	0.45989 (13)	0.0549 (5)	
C18	0.0576 (3)	0.85584 (18)	0.55318 (14)	0.0744 (6)	
H18	−0.0201	0.8204	0.5676	0.089*	
C19	0.0653 (4)	0.9221 (2)	0.62489 (19)	0.1005 (10)	
H19	−0.0066	0.9305	0.6874	0.121*	
C20	0.1766 (4)	0.9753 (2)	0.6051 (2)	0.1116 (12)	
H20	0.1817	1.0196	0.6540	0.134*	
C21	0.2820 (3)	0.9638 (2)	0.5125 (2)	0.0973 (9)	
H21	0.3574	1.0012	0.4982	0.117*	
C22	0.2758 (2)	0.89655 (16)	0.44051 (17)	0.0692 (6)	
H22	0.3481	0.8885	0.3782	0.083*	
C24	0.41689 (18)	0.70400 (12)	0.12498 (12)	0.0441 (4)	
H24	0.3373	0.6816	0.1038	0.053*	
C25	0.4743 (2)	0.79226 (13)	0.05688 (12)	0.0503 (4)	
H25	0.4203	0.8104	0.0045	0.060*	
C26	0.6241 (2)	0.70663 (14)	0.01974 (14)	0.0586 (5)	
C28	0.4838 (2)	0.88931 (14)	0.10429 (13)	0.0579 (5)	
C29	0.6204 (18)	0.8829 (16)	0.1303 (17)	0.074 (3)	0.46 (2)
H29	0.6975	0.8198	0.1139	0.088*	0.46 (2)
C30	0.6522 (16)	0.9628 (11)	0.1789 (9)	0.083 (3)	0.46 (2)
H30	0.7428	0.9527	0.1985	0.099*	0.46 (2)
C31	0.5410 (19)	1.0561 (10)	0.1953 (10)	0.086 (4)	0.46 (2)
H31	0.5545	1.1123	0.2277	0.103*	0.46 (2)
C32	0.4107 (19)	1.0685 (7)	0.1653 (8)	0.070 (3)	0.46 (2)
H32	0.3385	1.1341	0.1750	0.083*	0.46 (2)
C33	0.383 (2)	0.9867 (10)	0.1209 (19)	0.069 (4)	0.46 (2)
H33	0.2919	0.9983	0.1016	0.083*	0.46 (2)
C29A	0.5938 (18)	0.8965 (15)	0.1495 (16)	0.093 (5)	0.54 (2)
H29A	0.6760	0.8388	0.1516	0.111*	0.54 (2)
C30A	0.577 (2)	0.9938 (11)	0.1922 (12)	0.116 (5)	0.54 (2)
H30A	0.6534	1.0015	0.2203	0.139*	0.54 (2)
C31A	0.456 (3)	1.0783 (10)	0.1957 (10)	0.097 (4)	0.54 (2)
H31A	0.4515	1.1414	0.2265	0.117*	0.54 (2)
C32A	0.3408 (18)	1.0722 (7)	0.1540 (7)	0.078 (2)	0.54 (2)
H32A	0.2560	1.1296	0.1568	0.093*	0.54 (2)
C33A	0.3560 (17)	0.9759 (9)	0.1073 (16)	0.061 (2)	0.54 (2)
H33A	0.2804	0.9690	0.0777	0.074*	0.54 (2)
C34	0.62980 (19)	0.52261 (13)	0.08421 (12)	0.0457 (4)	
C35	0.7856 (2)	0.47680 (14)	0.04880 (15)	0.0582 (5)	
H35	0.8524	0.5189	0.0214	0.070*	
C36	0.8439 (2)	0.36877 (15)	0.05343 (16)	0.0638 (5)	
H36	0.9495	0.3384	0.0287	0.077*	
C37	0.7467 (2)	0.30656 (14)	0.09426 (14)	0.0581 (5)	
C38	0.5902 (2)	0.35246 (14)	0.12876 (14)	0.0580 (5)	
H38	0.5236	0.3102	0.1560	0.070*	
C39	0.5313 (2)	0.45959 (13)	0.12349 (13)	0.0511 (4)	
H39	0.4254	0.4895	0.1464	0.061*	
C40	0.9516 (3)	0.14970 (18)	0.0789 (2)	0.0958 (8)	
H40A	0.9674	0.0756	0.0928	0.144*	
H40B	1.0005	0.1817	0.1175	0.144*	
H40C	0.9957	0.1580	0.0088	0.144*	

### Atomic displacement parameters (Å^2^)

**Table:**

	*U*^11^	*U*^22^	*U*^33^	*U*^12^	*U*^13^	*U*^23^
O1	0.0501 (8)	0.1036 (12)	0.0806 (10)	−0.0273 (8)	0.0017 (7)	0.0018 (8)
O2	0.0529 (17)	0.059 (2)	0.0692 (18)	−0.0213 (15)	−0.0051 (12)	0.0002 (13)
O2A	0.078 (7)	0.068 (9)	0.129 (13)	−0.037 (7)	−0.020 (7)	−0.018 (7)
O3	0.1268 (15)	0.1068 (13)	0.1019 (13)	−0.0018 (11)	−0.0799 (12)	0.0268 (11)
O4	0.0999 (13)	0.1229 (15)	0.0954 (13)	−0.0543 (11)	−0.0568 (10)	0.0229 (10)
O5	0.0710 (10)	0.0672 (9)	0.0956 (11)	−0.0114 (7)	0.0269 (9)	0.0157 (8)
O6	0.0941 (12)	0.0454 (8)	0.1134 (13)	−0.0072 (8)	−0.0223 (10)	0.0046 (8)
N1	0.0351 (7)	0.0442 (7)	0.0410 (7)	−0.0052 (6)	−0.0103 (6)	0.0063 (5)
N23	0.0430 (8)	0.0810 (12)	0.0454 (9)	0.0024 (8)	−0.0147 (7)	0.0022 (8)
N27	0.0432 (8)	0.0445 (8)	0.0473 (8)	−0.0099 (6)	−0.0011 (6)	0.0022 (6)
C2	0.0402 (9)	0.0472 (9)	0.0494 (9)	0.0011 (7)	−0.0150 (7)	0.0013 (7)
C3	0.0487 (10)	0.0494 (10)	0.0584 (11)	0.0050 (8)	−0.0270 (9)	0.0000 (8)
C4	0.0549 (12)	0.0770 (14)	0.0889 (16)	0.0021 (10)	−0.0341 (11)	−0.0148 (11)
C5	0.0770 (17)	0.105 (2)	0.128 (2)	0.0033 (14)	−0.0611 (18)	−0.0376 (17)
C6	0.102 (2)	0.104 (2)	0.101 (2)	0.0252 (16)	−0.0688 (18)	−0.0399 (16)
C7	0.0826 (16)	0.0844 (15)	0.0590 (13)	0.0206 (12)	−0.0364 (12)	−0.0081 (11)
C8	0.0625 (12)	0.0552 (11)	0.0489 (10)	0.0092 (9)	−0.0261 (9)	0.0020 (8)
C9	0.0584 (11)	0.0621 (11)	0.0437 (10)	−0.0033 (9)	−0.0101 (8)	0.0124 (8)
C10	0.0385 (9)	0.0536 (10)	0.0412 (9)	−0.0069 (7)	−0.0075 (7)	0.0060 (7)
C11	0.0372 (9)	0.0574 (10)	0.0411 (9)	−0.0002 (7)	−0.0096 (7)	0.0020 (7)
C12	0.0415 (9)	0.0488 (9)	0.0428 (9)	−0.0023 (7)	−0.0139 (7)	0.0028 (7)
C13	0.0371 (8)	0.0427 (8)	0.0415 (9)	−0.0071 (7)	−0.0114 (7)	0.0039 (6)
C14	0.0475 (11)	0.0734 (13)	0.0558 (11)	−0.0188 (9)	−0.0076 (9)	0.0117 (10)
C15	0.075 (2)	0.081 (3)	0.085 (2)	−0.041 (2)	−0.0143 (18)	−0.001 (2)
C16	0.107 (3)	0.130 (4)	0.089 (3)	−0.048 (3)	−0.026 (2)	−0.026 (2)
C16A	0.121 (8)	0.101 (9)	0.139 (9)	−0.031 (7)	−0.057 (7)	−0.007 (7)
C15A	0.132 (10)	0.116 (9)	0.174 (11)	−0.065 (8)	−0.047 (8)	−0.016 (8)
C17	0.0513 (10)	0.0540 (10)	0.0495 (10)	0.0121 (8)	−0.0212 (8)	−0.0028 (8)
C18	0.0751 (14)	0.0781 (14)	0.0476 (11)	0.0200 (11)	−0.0164 (10)	−0.0062 (10)
C19	0.104 (2)	0.107 (2)	0.0609 (14)	0.0401 (17)	−0.0328 (14)	−0.0246 (14)
C20	0.119 (2)	0.101 (2)	0.103 (2)	0.0429 (19)	−0.071 (2)	−0.0508 (17)
C21	0.0903 (18)	0.0791 (16)	0.127 (2)	0.0109 (13)	−0.0622 (18)	−0.0333 (15)
C22	0.0651 (13)	0.0628 (12)	0.0764 (14)	0.0058 (10)	−0.0327 (11)	−0.0142 (10)
C24	0.0401 (9)	0.0455 (9)	0.0427 (9)	−0.0074 (7)	−0.0084 (7)	0.0030 (7)
C25	0.0552 (10)	0.0486 (10)	0.0417 (9)	−0.0101 (8)	−0.0085 (8)	0.0070 (7)
C26	0.0574 (11)	0.0522 (11)	0.0551 (11)	−0.0132 (8)	0.0016 (9)	0.0041 (8)
C28	0.0770 (13)	0.0501 (11)	0.0453 (10)	−0.0217 (10)	−0.0108 (9)	0.0124 (8)
C29	0.104 (6)	0.068 (5)	0.052 (5)	−0.040 (4)	−0.009 (4)	−0.005 (3)
C30	0.097 (6)	0.083 (6)	0.075 (4)	−0.034 (5)	−0.025 (5)	−0.005 (4)
C31	0.116 (9)	0.075 (7)	0.072 (5)	−0.048 (7)	−0.010 (6)	−0.001 (5)
C32	0.084 (7)	0.050 (4)	0.067 (5)	−0.019 (5)	−0.006 (5)	0.000 (3)
C33	0.103 (8)	0.040 (5)	0.056 (6)	−0.019 (4)	−0.008 (5)	0.000 (3)
C29A	0.130 (9)	0.091 (9)	0.091 (11)	−0.063 (7)	−0.056 (9)	0.030 (6)
C30A	0.157 (12)	0.097 (9)	0.150 (10)	−0.077 (10)	−0.097 (10)	0.045 (7)
C31A	0.163 (13)	0.068 (5)	0.096 (6)	−0.058 (7)	−0.066 (8)	0.012 (4)
C32A	0.097 (5)	0.055 (3)	0.083 (4)	−0.031 (4)	−0.018 (4)	0.011 (2)
C33A	0.085 (4)	0.044 (4)	0.052 (5)	−0.022 (3)	−0.008 (3)	0.002 (3)
C34	0.0476 (9)	0.0436 (9)	0.0423 (9)	−0.0094 (7)	−0.0078 (7)	−0.0015 (7)
C35	0.0462 (10)	0.0525 (11)	0.0701 (12)	−0.0128 (8)	−0.0066 (9)	0.0026 (9)
C36	0.0499 (11)	0.0547 (11)	0.0784 (14)	−0.0038 (9)	−0.0123 (10)	−0.0014 (9)
C37	0.0679 (12)	0.0454 (10)	0.0589 (11)	−0.0084 (9)	−0.0191 (9)	−0.0033 (8)
C38	0.0672 (12)	0.0496 (10)	0.0563 (11)	−0.0233 (9)	−0.0068 (9)	−0.0030 (8)
C39	0.0472 (10)	0.0510 (10)	0.0510 (10)	−0.0136 (8)	−0.0047 (8)	−0.0061 (8)
C40	0.1015 (19)	0.0581 (14)	0.116 (2)	0.0125 (13)	−0.0417 (16)	0.0010 (13)

### Geometric parameters (Å, °)

**Table:**

O1—C14	1.193 (2)	C15A—H15C	0.97
O2—C14	1.340 (6)	C15A—H15D	0.97
O2—C15	1.440 (7)	C17—C22	1.378 (3)
O2A—C14	1.32 (2)	C17—C18	1.384 (3)
O2A—C15A	1.49 (3)	C18—C19	1.376 (4)
O3—N23	1.202 (2)	C18—H18	0.93
O4—N23	1.202 (2)	C19—C20	1.355 (4)
O5—C26	1.203 (2)	C19—H19	0.93
O6—C37	1.369 (2)	C20—C21	1.373 (4)
O6—C40	1.410 (3)	C20—H20	0.93
N1—C13	1.4552 (19)	C21—C22	1.381 (3)
N1—C10	1.4648 (19)	C21—H21	0.93
N1—C2	1.471 (2)	C22—H22	0.93
N23—C12	1.501 (2)	C24—C25	1.568 (2)
N27—C26	1.356 (2)	C24—H24	0.98
N27—C34	1.411 (2)	C25—C28	1.488 (3)
N27—C24	1.475 (2)	C25—C26	1.519 (2)
C2—C3	1.493 (2)	C25—H25	0.98
C2—H2A	0.97	C28—C33	1.357 (10)
C2—H2B	0.97	C28—C29A	1.362 (9)
C3—C4	1.370 (3)	C28—C29	1.395 (11)
C3—C8	1.390 (3)	C28—C33A	1.405 (10)
C4—C5	1.376 (3)	C29—C30	1.401 (11)
C4—H4	0.93	C29—H29	0.93
C5—C6	1.365 (4)	C30—C31	1.360 (12)
C5—H5	0.93	C30—H30	0.93
C6—C7	1.366 (4)	C31—C32	1.356 (11)
C6—H6	0.93	C31—H31	0.93
C7—C8	1.381 (3)	C32—C33	1.370 (11)
C7—H7	0.93	C32—H32	0.93
C8—C9	1.482 (3)	C33—H33	0.93
C9—C10	1.547 (2)	C29A—C30A	1.380 (11)
C9—H9A	0.97	C29A—H29A	0.93
C9—H9B	0.97	C30A—C31A	1.344 (11)
C10—C14	1.532 (3)	C30A—H30A	0.93
C10—C11	1.563 (2)	C31A—C32A	1.375 (9)
C11—C17	1.511 (2)	C31A—H31A	0.93
C11—C12	1.524 (2)	C32A—C33A	1.398 (10)
C11—H11	0.98	C32A—H32A	0.93
C12—C13	1.532 (2)	C33A—H33A	0.93
C12—H12	0.98	C34—C35	1.373 (2)
C13—C24	1.523 (2)	C34—C39	1.377 (2)
C13—H13	0.98	C35—C36	1.380 (3)
C15—C16	1.464 (5)	C35—H35	0.93
C15—H15A	0.97	C36—C37	1.365 (3)
C15—H15B	0.97	C36—H36	0.93
C16—H16A	0.96	C37—C38	1.378 (3)
C16—H16B	0.96	C38—C39	1.372 (2)
C16—H16C	0.96	C38—H38	0.93
C16A—C15A	1.439 (10)	C39—H39	0.93
C16A—H16D	0.96	C40—H40A	0.96
C16A—H16E	0.96	C40—H40B	0.96
C16A—H16F	0.96	C40—H40C	0.96
			
C14—O2—C15	116.3 (4)	C22—C17—C18	117.98 (19)
C14—O2A—C15A	116.2 (16)	C22—C17—C11	123.72 (17)
C37—O6—C40	117.86 (18)	C18—C17—C11	118.28 (19)
C13—N1—C10	111.04 (12)	C19—C18—C17	120.9 (3)
C13—N1—C2	113.18 (12)	C19—C18—H18	119.6
C10—N1—C2	115.31 (12)	C17—C18—H18	119.6
O4—N23—O3	123.91 (19)	C20—C19—C18	120.5 (3)
O4—N23—C12	119.47 (16)	C20—C19—H19	119.8
O3—N23—C12	116.62 (19)	C18—C19—H19	119.8
C26—N27—C34	131.40 (14)	C19—C20—C21	119.8 (3)
C26—N27—C24	94.69 (13)	C19—C20—H20	120.1
C34—N27—C24	131.58 (13)	C21—C20—H20	120.1
N1—C2—C3	112.50 (13)	C20—C21—C22	119.9 (3)
N1—C2—H2A	109.1	C20—C21—H21	120.0
C3—C2—H2A	109.1	C22—C21—H21	120.0
N1—C2—H2B	109.1	C17—C22—C21	120.9 (2)
C3—C2—H2B	109.1	C17—C22—H22	119.6
H2A—C2—H2B	107.8	C21—C22—H22	119.6
C4—C3—C8	120.07 (18)	N27—C24—C13	116.31 (13)
C4—C3—C2	124.54 (18)	N27—C24—C25	86.97 (11)
C8—C3—C2	115.39 (16)	C13—C24—C25	115.86 (14)
C3—C4—C5	119.6 (2)	N27—C24—H24	111.8
C3—C4—H4	120.2	C13—C24—H24	111.8
C5—C4—H4	120.2	C25—C24—H24	111.8
C6—C5—C4	120.2 (3)	C28—C25—C26	116.96 (16)
C6—C5—H5	119.9	C28—C25—C24	119.41 (14)
C4—C5—H5	119.9	C26—C25—C24	84.88 (12)
C7—C6—C5	120.8 (2)	C28—C25—H25	111.1
C7—C6—H6	119.6	C26—C25—H25	111.1
C5—C6—H6	119.6	C24—C25—H25	111.1
C6—C7—C8	119.6 (2)	O5—C26—N27	131.23 (17)
C6—C7—H7	120.2	O5—C26—C25	135.39 (17)
C8—C7—H7	120.2	N27—C26—C25	93.38 (13)
C7—C8—C3	119.6 (2)	C33—C28—C29A	103.8 (8)
C7—C8—C9	124.6 (2)	C33—C28—C29	114.0 (11)
C3—C8—C9	115.77 (16)	C29A—C28—C33A	120.6 (8)
C8—C9—C10	112.11 (15)	C29—C28—C33A	130.7 (9)
C8—C9—H9A	109.2	C33—C28—C25	129.7 (8)
C10—C9—H9A	109.2	C29A—C28—C25	126.3 (8)
C8—C9—H9B	109.2	C29—C28—C25	116.2 (7)
C10—C9—H9B	109.2	C33A—C28—C25	112.9 (6)
H9A—C9—H9B	107.9	C28—C29—C30	126.0 (14)
N1—C10—C14	111.71 (14)	C28—C29—H29	117.0
N1—C10—C9	111.98 (13)	C30—C29—H29	117.0
C14—C10—C9	103.09 (14)	C31—C30—C29	115.1 (11)
N1—C10—C11	106.09 (12)	C31—C30—H30	122.5
C14—C10—C11	109.45 (14)	C29—C30—H30	122.5
C9—C10—C11	114.63 (14)	C32—C31—C30	121.1 (10)
C17—C11—C12	112.23 (15)	C32—C31—H31	119.5
C17—C11—C10	117.47 (13)	C30—C31—H31	119.5
C12—C11—C10	103.54 (12)	C31—C32—C33	121.5 (9)
C17—C11—H11	107.7	C31—C32—H32	119.2
C12—C11—H11	107.7	C33—C32—H32	119.2
C10—C11—H11	107.7	C28—C33—C32	122.1 (10)
N23—C12—C11	110.10 (14)	C28—C33—H33	118.9
N23—C12—C13	112.54 (13)	C32—C33—H33	118.9
C11—C12—C13	105.38 (12)	C28—C29A—C30A	116.5 (11)
N23—C12—H12	109.6	C28—C29A—H29A	121.7
C11—C12—H12	109.6	C30A—C29A—H29A	121.7
C13—C12—H12	109.6	C31A—C30A—C29A	124.3 (10)
N1—C13—C24	113.69 (13)	C31A—C30A—H30A	117.9
N1—C13—C12	106.24 (12)	C29A—C30A—H30A	117.9
C24—C13—C12	113.66 (13)	C30A—C31A—C32A	120.5 (9)
N1—C13—H13	107.7	C30A—C31A—H31A	119.8
C24—C13—H13	107.7	C32A—C31A—H31A	119.8
C12—C13—H13	107.7	C31A—C32A—C33A	117.1 (9)
O1—C14—O2A	121.8 (8)	C31A—C32A—H32A	121.4
O1—C14—O2	124.0 (3)	C33A—C32A—H32A	121.4
O1—C14—C10	122.06 (19)	C32A—C33A—C28	120.9 (10)
O2A—C14—C10	113.8 (8)	C32A—C33A—H33A	119.5
O2—C14—C10	113.1 (3)	C28—C33A—H33A	119.5
O2—C15—C16	113.0 (5)	C35—C34—C39	119.39 (16)
O2—C15—H15A	109.0	C35—C34—N27	120.06 (15)
C16—C15—H15A	109.0	C39—C34—N27	120.54 (15)
O2—C15—H15B	109.0	C34—C35—C36	120.61 (17)
C16—C15—H15B	109.0	C34—C35—H35	119.7
H15A—C15—H15B	107.8	C36—C35—H35	119.7
C15—C16—H16A	109.5	C37—C36—C35	120.02 (18)
C15—C16—H16B	109.5	C37—C36—H36	120.0
H16A—C16—H16B	109.5	C35—C36—H36	120.0
C15—C16—H16C	109.5	C36—C37—O6	125.13 (18)
H16A—C16—H16C	109.5	C36—C37—C38	119.31 (17)
H16B—C16—H16C	109.5	O6—C37—C38	115.55 (17)
C15A—C16A—H16D	109.5	C39—C38—C37	120.95 (17)
C15A—C16A—H16E	109.5	C39—C38—H38	119.5
H16D—C16A—H16E	109.5	C37—C38—H38	119.5
C15A—C16A—H16F	109.5	C38—C39—C34	119.69 (16)
H16D—C16A—H16F	109.5	C38—C39—H39	120.2
H16E—C16A—H16F	109.5	C34—C39—H39	120.2
C16A—C15A—O2A	108 (2)	O6—C40—H40A	109.5
C16A—C15A—H15C	110.0	O6—C40—H40B	109.5
O2A—C15A—H15C	110.0	H40A—C40—H40B	109.5
C16A—C15A—H15D	110.0	O6—C40—H40C	109.5
O2A—C15A—H15D	110.0	H40A—C40—H40C	109.5
H15C—C15A—H15D	108.4	H40B—C40—H40C	109.5
			
C13—N1—C2—C3	84.31 (17)	C19—C20—C21—C22	−1.2 (4)
C10—N1—C2—C3	−45.06 (19)	C18—C17—C22—C21	0.5 (3)
N1—C2—C3—C4	−130.67 (18)	C11—C17—C22—C21	178.70 (18)
N1—C2—C3—C8	49.8 (2)	C20—C21—C22—C17	0.6 (3)
C8—C3—C4—C5	1.6 (3)	C26—N27—C24—C13	−119.64 (16)
C2—C3—C4—C5	−177.9 (2)	C34—N27—C24—C13	76.7 (2)
C3—C4—C5—C6	0.0 (4)	C26—N27—C24—C25	−2.18 (14)
C4—C5—C6—C7	−1.0 (4)	C34—N27—C24—C25	−165.88 (17)
C5—C6—C7—C8	0.5 (4)	N1—C13—C24—N27	−58.31 (18)
C6—C7—C8—C3	1.1 (3)	C12—C13—C24—N27	179.98 (13)
C6—C7—C8—C9	−177.93 (19)	N1—C13—C24—C25	−158.34 (13)
C4—C3—C8—C7	−2.1 (3)	C12—C13—C24—C25	79.95 (18)
C2—C3—C8—C7	177.42 (16)	N27—C24—C25—C28	−116.22 (17)
C4—C3—C8—C9	176.97 (17)	C13—C24—C25—C28	1.7 (2)
C2—C3—C8—C9	−3.5 (2)	N27—C24—C25—C26	1.95 (13)
C7—C8—C9—C10	134.24 (19)	C13—C24—C25—C26	119.82 (15)
C3—C8—C9—C10	−44.8 (2)	C34—N27—C26—O5	−13.6 (4)
C13—N1—C10—C14	111.95 (15)	C24—N27—C26—O5	−177.4 (2)
C2—N1—C10—C14	−117.64 (15)	C34—N27—C26—C25	165.99 (17)
C13—N1—C10—C9	−132.99 (15)	C24—N27—C26—C25	2.25 (15)
C2—N1—C10—C9	−2.58 (19)	C28—C25—C26—O5	−62.0 (3)
C13—N1—C10—C11	−7.25 (17)	C24—C25—C26—O5	177.5 (3)
C2—N1—C10—C11	123.17 (14)	C28—C25—C26—N27	118.39 (16)
C8—C9—C10—N1	47.6 (2)	C24—C25—C26—N27	−2.12 (14)
C8—C9—C10—C14	167.77 (15)	C26—C25—C28—C33	161.5 (15)
C8—C9—C10—C11	−73.36 (19)	C24—C25—C28—C33	−98.6 (15)
N1—C10—C11—C17	−102.52 (16)	C26—C25—C28—C29A	−24.7 (12)
C14—C10—C11—C17	136.81 (16)	C24—C25—C28—C29A	75.1 (12)
C9—C10—C11—C17	21.6 (2)	C26—C25—C28—C29	−13.9 (12)
N1—C10—C11—C12	21.79 (16)	C24—C25—C28—C29	86.0 (12)
C14—C10—C11—C12	−98.87 (15)	C26—C25—C28—C33A	161.0 (10)
C9—C10—C11—C12	145.91 (14)	C24—C25—C28—C33A	−99.1 (10)
O4—N23—C12—C11	−47.9 (2)	C33—C28—C29—C30	6(3)
O3—N23—C12—C11	132.67 (18)	C29A—C28—C29—C30	−37 (6)
O4—N23—C12—C13	69.3 (2)	C33A—C28—C29—C30	9(3)
O3—N23—C12—C13	−110.10 (19)	C25—C28—C29—C30	−177.5 (17)
C17—C11—C12—N23	−138.51 (14)	C28—C29—C30—C31	−4(3)
C10—C11—C12—N23	93.83 (15)	C29—C30—C31—C32	−0.4 (19)
C17—C11—C12—C13	99.89 (15)	C30—C31—C32—C33	3(2)
C10—C11—C12—C13	−27.77 (16)	C29A—C28—C33—C32	6(3)
C10—N1—C13—C24	−136.12 (14)	C29—C28—C33—C32	−4(3)
C2—N1—C13—C24	92.35 (16)	C33A—C28—C33—C32	−178 (9)
C10—N1—C13—C12	−10.37 (17)	C25—C28—C33—C32	−179.4 (12)
C2—N1—C13—C12	−141.90 (13)	C31—C32—C33—C28	0(3)
N23—C12—C13—N1	−95.83 (16)	C33—C28—C29A—C30A	−3(2)
C11—C12—C13—N1	24.16 (17)	C29—C28—C29A—C30A	137 (10)
N23—C12—C13—C24	29.9 (2)	C33A—C28—C29A—C30A	−4(2)
C11—C12—C13—C24	149.93 (14)	C25—C28—C29A—C30A	−177.5 (11)
C15A—O2A—C14—O1	−8(2)	C28—C29A—C30A—C31A	3(3)
C15A—O2A—C14—O2	96 (3)	C29A—C30A—C31A—C32A	−1(2)
C15A—O2A—C14—C10	−170.6 (15)	C30A—C31A—C32A—C33A	−1(2)
C15—O2—C14—O1	1.2 (5)	C31A—C32A—C33A—C28	1(2)
C15—O2—C14—O2A	−92 (2)	C33—C28—C33A—C32A	−2(5)
C15—O2—C14—C10	170.9 (3)	C29A—C28—C33A—C32A	2(3)
N1—C10—C14—O1	−164.28 (18)	C29—C28—C33A—C32A	−10 (3)
C9—C10—C14—O1	75.3 (2)	C25—C28—C33A—C32A	176.5 (14)
C11—C10—C14—O1	−47.1 (2)	C26—N27—C34—C35	29.8 (3)
N1—C10—C14—O2A	−1.3 (10)	C24—N27—C34—C35	−172.06 (17)
C9—C10—C14—O2A	−121.7 (10)	C26—N27—C34—C39	−151.74 (19)
C11—C10—C14—O2A	115.9 (10)	C24—N27—C34—C39	6.4 (3)
N1—C10—C14—O2	25.8 (3)	C39—C34—C35—C36	−0.8 (3)
C9—C10—C14—O2	−94.6 (3)	N27—C34—C35—C36	177.66 (17)
C11—C10—C14—O2	143.0 (2)	C34—C35—C36—C37	−0.6 (3)
C14—O2—C15—C16	79.2 (4)	C35—C36—C37—O6	−178.58 (19)
C14—O2A—C15A—C16A	−107.7 (16)	C35—C36—C37—C38	1.3 (3)
C12—C11—C17—C22	−28.3 (2)	C40—O6—C37—C36	5.4 (3)
C10—C11—C17—C22	91.6 (2)	C40—O6—C37—C38	−174.5 (2)
C12—C11—C17—C18	149.89 (16)	C36—C37—C38—C39	−0.6 (3)
C10—C11—C17—C18	−90.27 (19)	O6—C37—C38—C39	179.23 (17)
C22—C17—C18—C19	−1.2 (3)	C37—C38—C39—C34	−0.7 (3)
C11—C17—C18—C19	−179.44 (18)	C35—C34—C39—C38	1.4 (3)
C17—C18—C19—C20	0.6 (4)	N27—C34—C39—C38	−177.02 (16)
C18—C19—C20—C21	0.5 (4)		
